# Diving Hazards Unmasked: Estimating Infection Risk from Pathogen Exposure

**Published:** 2006-05

**Authors:** Nancy Bazilchuk

Most recreational water quality standards are aimed at protecting beachgoers against accidental ingestion of or skin contact with water contaminated by fecal material. But the increased popularity of water sports such as kayaking, surfing, and diving, which often occur far from regulated bathing beaches, raises the question of the water-related health risks these sports entail. Now two researchers at the National Institute of Public Health and the Environment in the Netherlands have attempted to answer this question for divers **[*EHP* 114:712–717; Schijven and Husman]**. The study is the first to establish estimates of how much water divers swallow, figures that can be used in calculating health risks involved with waterborne pathogen exposure during diving.

The researchers used detailed questionnaires to ask occupational and sport divers about the number and duration of dives they made in ocean, coastal, and freshwater areas; whether a known pollution source was nearby; the type of diving mask worn (which affects the amount of water swallowed); and the amount of water typically swallowed per dive. Five equivalents enabled divers to estimate how much water they swallowed: nothing, a few drops (an average of 2.75 milliliters [mL]), a shot glass (25 mL), a coffee cup (100 mL), or a soda glass (190 mL). The questionnaire also asked respondents to detail past health complaints, including diarrhea, vomiting, nausea, and eye, skin, and ear problems.

Then the researchers calculated the risk of infection per dive and per year based on the volume of swallowed water reported and pathogen concentrations. *Campylobacter jejuni* and enteroviruses were used for the analysis; concentrations for these organisms in different kinds of surface waters were taken from the literature, and concentration distributions constructed.

The infection risks for *C. jejuni* were generally an order of magnitude higher than those for enteroviruses. For occupational divers, the greatest per-dive mean risk of infection was calculated at 2.8% in coastal waters near a sewage discharge. For sport divers wearing ordinary diving masks, the greatest mean risk was seen in freshwater recreational waters, where there was a 1.5% per-dive risk and a 25% per-year risk of getting an infection. The risk was about 10 times lower when sport divers wore full face masks.

Although occupational divers usually have the protection of a full face mask or diving helmet, they are far more likely than sport divers to dive in contaminated conditions—for example, to assess damage to underwater sewage pipes. They also tend to stay underwater longer. Thus, the chance for exposure goes up.

These relatively high infection risks may explain why 80% of the divers surveyed reported at least one of the health complaints listed on the questionnaire during the course of one year. The authors recommend that divers wear full face masks or helmets when diving in potentially contaminated waters, and that they stay informed about fecal contamination in diving areas.

## Figures and Tables

**Figure:**
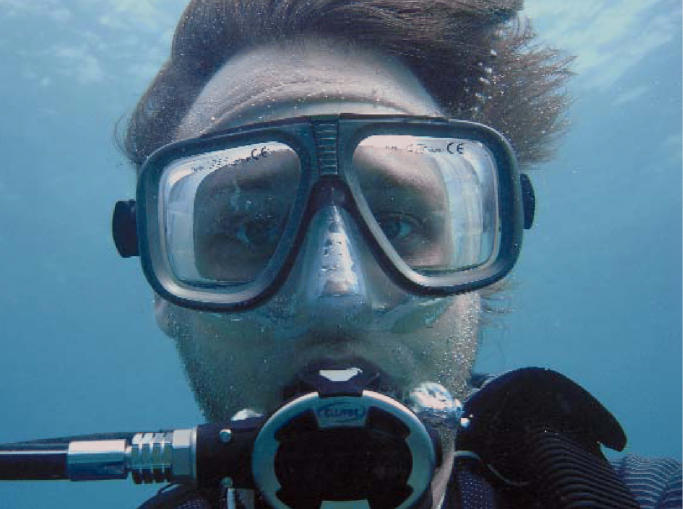
What lies beneath. A new study estimates divers’ risk of developing infections when diving in fecal-contaminated waters.

